# P-949. Discharge Delays and Costs Associated with Outpatient Parenteral Antimicrobial Therapy for Multi-drug-resistant Organisms: Opportunities for Improvement

**DOI:** 10.1093/ofid/ofaf695.1152

**Published:** 2026-01-11

**Authors:** Stormmy Boettcher, Rachel M Kenney, Nathan Everson, Surafel MULUGETA, Geehan Suleyman, Anita Shallal, Michael P Veve

**Affiliations:** Wayne State University/Henry Ford Hospital, Plymouth, MI; Henry Ford Hospital, Detroit, Michigan; Henry Ford Hospital, Detroit, Michigan; Henry Ford Health, Detroit, Michigan; Henry Ford Health, Detroit, Michigan; Henry Ford Hospital, Detroit, Michigan; Eugene Applebaum College of Pharmacy and Health Sciences, Detroit, MI

## Abstract

**Background:**

The coordination of outpatient parenteral antimicrobial therapy (OPAT) transitions of care is challenging in patients with multi-drug-resistant organisms (MDRO) due to complexity of care. The study purpose was to describe barriers and medication costs associated with OPAT utilizing novel therapies for MDRO.

**Table 1.**
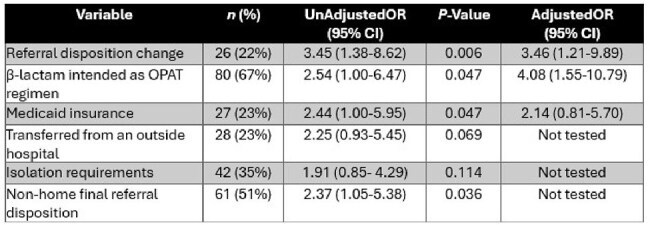
Variables associated with receiving a modified outpatient parenteral antimicrobial therapy regimen. Abbreviations: OPAT, outpatient parenteral antimicrobial therapy Hosmer-Lemeshow Goodness of Fit testing: Chi-square, 0.030, P=0.985

**Methods:**

IRB approved, retrospective cohort of hospitalized adults infected with MDRO that were medically stable for discharge (MSDC) with an intended OPAT for cefiderocol, ceftazidime/avibactam, ceftolozane/tazobactam, eravacycline, meropenem/vaborbactam, or tigecycline from 01/01/2017-03/31/2025. Cohorts included patients who received an intended OPAT regimen or a modified OPAT regimen, defined as transition to alternative intravenous (IV)/oral therapy, in-hospital completion of IV therapy, or in-hospital death. Secondary outcomes included post-MSDC medication costs, excess hospitalization, and opportunities for oral-switch therapy.

**Results:**

120 patients were included. 29% had a modified OPAT regimen; 46% completed therapy inpatient and 23% were transitioned to oral therapy. Of the total population, the majority were men (58%) and had Medicare (53%). Intra-abdominal infections were common (38%), and most organisms were carbapenem-resistant (89%). β-lactams were the most intended OPAT regimen (67%). Patients with a modified OPAT regimen had significantly higher medication costs ($4828 [$1209-$18066] vs $1975 [$494-$4872], P< 0.001), more frequently experienced discharge delays ≥ 1 day (89% vs 66%, P=0.011) and prolonged length of stay (LOS) (20 [14-46] vs 13 [7-27], P=0.023), and more commonly required a change in discharge referral disposition (40% vs 16%, P=0.006) when compared to those who received an intended OPAT regimen. An oral-switch therapy opportunity was identified in 40% of patients. After adjusting for Medicaid, referral disposition changes and β-lactam therapy were associated with an increased odds of receiving a modified OPAT regimen (Table 1).

**Conclusion:**

Modified OPAT regimens were common and associated with increased costs, prolonged LOS, and discharge delays in patients with MRDO infections. Findings support use of oral-switch therapy and improved care coordination.

**Disclosures:**

All Authors: No reported disclosures

